# Relationship Between the Carbonation Depth and Microstructure of Concrete Under Freeze–Thaw Conditions

**DOI:** 10.3390/ma17246191

**Published:** 2024-12-18

**Authors:** Shuhua Zhang, Guangrong Tan, Zhiqiang Qi, Bin Tian, Jijun Cao, Bofu Chen

**Affiliations:** 1 The Seventh Geological Brigade of Hubei Geological Bureau, Yichang 443000, China; shuhuazhang2022@163.com (S.Z.);; 2 Key Experiment of Geological Resources and Geological Engineering in Yichang, Yichang 443000, China; 3 College of Hydraulic and Environmental Engineering, Three Gorges University, Yichang 443000, China

**Keywords:** concrete, freeze–thaw, carbonization, microstructure, carbonation depth

## Abstract

Concrete structures in cold regions are affected by freeze–thaw cycles (FTCs) and carbonation, which lead to the premature failure of concrete structures. The carbonation depth, relative dynamic elastic modulus (RDEM), compressive strength, porosity, and pore size distribution of concrete under FTC conditions were tested through an accelerated carbonation experiment to study the carbonation performance evolution. The freeze–thaw effect mechanism on concrete carbonation was further analyzed via the obtained relationship between carbonation depth and pore structure. The results showed that the FTC, as a powerful source of concrete damage, accelerates the carbonation reaction. Carbonization products fill some microcracks caused by the freeze–thaw process, improve the compressive strength and dynamic elastic modulus, and alleviate the damage to concrete caused by the FTC. After carbonization under freeze–thaw damage conditions, the content of macropores with d > 1000 nm decreases, while the content of transition pores with d ≤ 10 nm increases, which is the direct reason for the decrease in porosity and the improvement in strength. Therefore, the carbonation durability of concrete under freeze–thaw conditions can be improved by controlling the content of macropores with d > 1000 nm and increasing the content of transition pores with a pore size of 10 nm ≤ d < 100 nm. In addition, the relationship between carbonation depth and pore structure under freeze–thaw conditions was established, and the research results can provide a reference for the study of the carbonation performance of concrete under freeze–thaw conditions.

## 1. Introduction

CO_2_ is everywhere in the atmospheric environment, and concrete structures are inevitably affected [1,2,3]. In fact, the carbonization of concrete is a precondition of steel corrosion and, thus, is one of the key problems that affects the durability of reinforced concrete structures. In this scenario, CO_2_ in the atmosphere intrudes into the concrete and reacts with the hydration product Ca(OH)_2_, which leads to a decrease in the alkalinity of the concrete around the steel bar and makes the steel bar in the concrete lose its passivation and protection function [4]. When the carbonation depth exceeds the thickness of the protective layer, the concrete no longer protects the steel bar from the action of air and water, the alkaline environment around the steel bar disappears, the passive film is destroyed, the steel bar rusts, and the concrete cracks [5,6]. Therefore, the carbonation depth of concrete is crucial to ensuring the safety and quality of construction projects [7,8] and must be predicted when studying the durability of reinforced concrete structures [9,10]. Furthermore, given that it is mainly environmental factors that affect concrete carbonation, concrete carbonation models should also consider the influence of environmental conditions [11]. The highest and lowest temperature records of 303 weather stations in China from 1961 to 2003 show that the extreme average low temperature in northeast China reached −30 °C from December to February of the following year [12]. According to the meteorological bureau of Xinjiang Uygur Autonomous Region in China, the lowest temperature in winter in Xinjiang is −43.3 °C [13]. Concrete structures in cold regions of China inevitably suffer from freeze–thaw cycle (FTC) damage during their service [14,15,16,17], and FTC damage is one of the main factors leading to the deterioration of reinforced concrete structures in cold areas, seriously affecting their service life [18,19]. Moreover, the FTC and carbonation are not simple superpositions of two separate actions but one complex coupling effect. Concrete structures in cold areas are subjected to the alternating action of FTC and carbonation, and so the carbonation depth of concrete is bound to be affected by the FTC.

At present, three main types of models can predict the carbonation depth of concrete: theoretical models [20,21], empirical models [22,23], and semitheoretical/semiempirical models [10,24,25]. Tan et al. [26] used X-ray diffraction and scanning electron microscopy (SEM) to analyze the influence of the microstructure of polypropylene-fiber-reinforced machine-made sand concrete on carbonation resistance, and they established a prediction model of carbonation depth. Geng et al. [27] established a prediction model of the carbonation depth of recycled concrete through regression analysis of experimental data, which can be used to predict the carbonation depth of recycled concrete structures in atmospheric environments. Through a rapid carbonation test, Zhang et al. studied the influence of the carbonation time and fly ash content on the carbonation performance of concrete and obtained a prediction model of the carbonation depth of fly ash concrete through curve fitting of the test data [28]. In accordance with the practical carbonation prediction model and nonlinear regression technology based on the least square method, a multifactor calculation model of concrete carbonation depth in a standard carbonation environment was established [29]. The carbonation microstructure and porosity of concrete change in FTC and carbonation environments, affecting the carbonation rate and carbonation depth [30,31]. Substantial research has been conducted on the durability of concrete under the action of single factors, such as FTC and carbonation [32,33]. For instance, some scholars performed a systematic study on a prediction model of the carbonation depth of concrete under single carbonation [2,3], and many conclusions and empirical formulas have reached consensus in academic circles and have been widely used and verified in practical projects. However, most concrete structures in practical engineering are affected by various environmental factors at the same time, and the failure of such concrete structures in actual service is complex and serious. Therefore, the research conclusions and empirical formulas obtained under single environmental conditions have certain limitations. In an actual service environment, the existence of the FTC and carbonation makes the durability evolution of concrete more complicated, particularly since the FTC changes the pore structure, which has a more complicated and unpredictable effect on carbonation.

In view of the importance of the carbonation depth for evaluating the carbonation resistance of concrete and predicting the corrosion initiation time of steel bars, models of the relationship between carbonation depth and micropore structure must be studied under FTC damage conditions. In this study, a carbonation experiment is conducted on concrete in an FTC environment. Using the carbonation depth, RDEM, compressive strength, porosity, and pore size distribution (PSD) as durability indicators, the influence of the FTC on the carbonation depth and carbonation performance of concrete is studied, and the evolution law of the carbonation resistance of concrete in an FTC environment is analyzed from macro and micro perspectives.

## 2. Experimental Program

**Figure 1:**
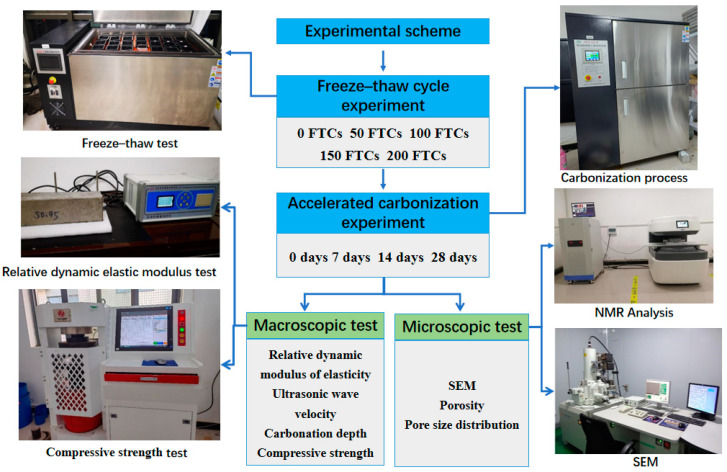
Flow diagram of the experiment.

The experimental program includes an FTC and accelerated carbonization. The macroscopic parameters tested include the carbonation depth, RDEM, and compressive strength, while the microscopic parameters include porosity and PSD. The test procedure is shown in Figure 1.

### 2.1. Materials

The cement used was ordinary Portland cement P.O. 42.5, which is produced by Gezhouba Cement Factory in China, with a density of 3150 kg/m^3^ and a specific surface area of 342 m^2^/kg. Grade II fly ash produced by Yichang Chengkun Building Materials Co., Ltd., Yichang, China. is adopted, with fineness of 8.7% and loss on ignition of 7%. Sand with a fineness modulus of 2.78 was used as fine aggregate, produced in Yichang, China, with a silt content of 0.81%. Two-graded aggregate is selected for concrete, which is divided into two grades: 5~20 mm and 20~40 mm, of which small stone and medium stone are in a 1:1 ratio of artificial crushed stone aggregate, requirements of the composite fuller grading curve [34]. Additives, such as a highly efficient naphthalene water-reducing agent and an air-entraining agent, were included. The select MH-JS-102 type water-reducing agent is produced by Hunan Minghuang Technology Development Co., Ltd., Changsha, China, and the air-entraining agent is Q8122AE high-efficiency air-entraining agent produced by China Shaanxi Qinfen Building Materials Co., Ltd., Xi’an, China. The mixing water is taken from ordinary tap water in Yichang City, Hubei Province. After testing, the air content of fresh concrete is 5%. The mix proportion of the concrete specimens is shown in Table 1.

### 2.2. Specimen Preparation

In a research experiment on the carbonation durability of concrete in rockfill dams under FTC damage conditions, a rapid carbonation process was conducted after the FTC. During the FTC, the concrete specimens were basically in a water-saturated state; given that the solubility of CO_2_ in water is low and the moisture content of the specimen was high, this meant that CO_2_ could not invade the interior of the concrete to undergo a carbonation reaction. Meanwhile, considering the particularity of the actual service situation of concrete in extremely cold and cold regions, concrete in water level fluctuation zones generally undergoes an FTC in cold winter and spring. In hot summer and autumn, when the reservoir water level drops, the water content of the original FTC site decreases under solar radiation, and carbonation reactions occur due to the influence of CO_2_ in the atmosphere. Thus, an FTC followed by carbonation could simulate the effect of the FTC and carbonation on the durability of concrete within a year more accurately. In addition, before carbonation, concrete must be dried in a drying oven for 48 h at 60 °C to exclude the effect of internal water on carbonation. The carbonation process was performed with an ambient temperature of 20 °C. The carbonization test employed prismatic specimens with side lengths of 100 mm × 100 mm × 400 mm to test the RDEM and 100 mm × 100 mm × 100 mm cubic specimens to test the compressive strength and carbonization depth.

### 2.3. Freeze–Thaw Process and Accelerated Carbonization Process

**Figure 2:**
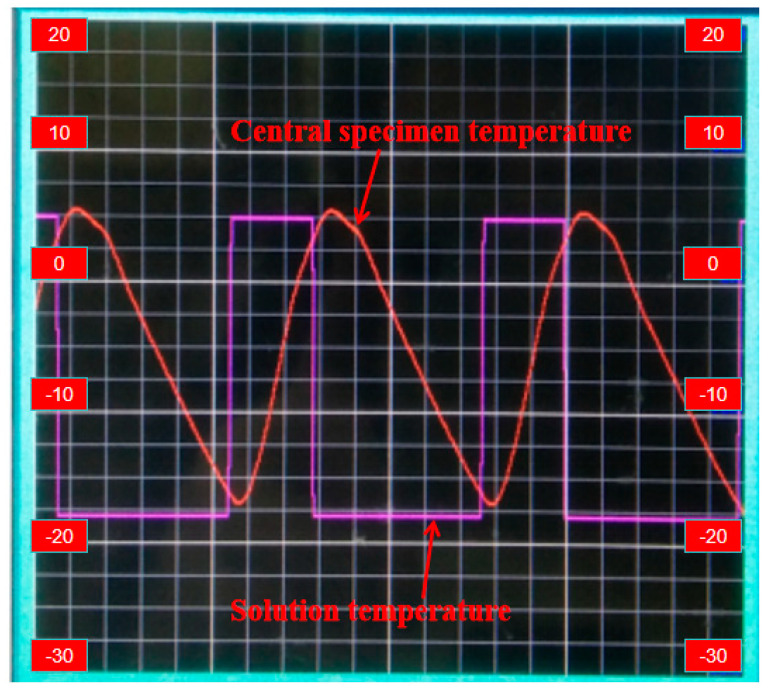
Real-time curve display of FTC.

FTC and carbonation tests were conducted in accordance with the operating procedures in the “Standard of Test Methods for Long-Term Performance and Durability of Ordinary Concrete” (GB/T50082-2009) [35]. The poured concrete specimens were cured in a standard curing room for 24 days, then soaked in water for 4 days until they were saturated with water and then taken out. Then, the specimens were dried with a rag, and the initial state (compressive strength, dynamic elastic modulus, pore structure) was tested before the freeze–thaw process. In the process of the FTC test, the parameters of the FTC tester are set: the center temperature of the specimen is (−17 ± 2) °C and (5 ± 2) °C, the FTC lasts no more than 4 h at a time, and the melting time is not less than 1/4 of the whole FTC. The real-time curve during the FTC is shown in Figure 2. After 50, 100, 150, and 200 FTCs, the concrete specimen was taken out to be tested, the surface moisture was dried, and the specimen was placed in a drying oven. The drying oven temperature was set to 60 °C, and carbonization commenced after 48 h of drying.

Concrete carbonation was conducted using an HTX-12X microcomputer concrete carbonation test box produced by Suzhou Donghua Test Instrument Co., Ltd., Suzhou, China. The dried specimen was placed on the bracket of the concrete carbonization test box, where the distance between the specimens should be greater than 50 mm. The sealing of the carbonization test box was checked before the switch was turned on. Carbonation was started when the CO_2_ concentration inside the carbonization box was steady at (20 ± 1)%, the temperature between the carbonation boxes was (20 ± 2) °C, and the relative humidity was controlled to (70 ± 2)%. After the carbonization test began, the parameters on the display of the carbonization box were checked every 4 h and adjusted in time to ensure that the temperature, humidity, and CO_2_ concentration in the carbonization box were within the set ranges. On the 7th, 14th, and 28th days, concrete specimens were taken out for measurement of the ultrasonic wave velocity, compressive strength, and carbonation depth.

### 2.4. Macro and Micro Test Program

The RDEM and compressive strength were tested in accordance with the “Standard for Test Methods of Long-Term Performance and Durability of Ordinary Concrete” [35]. After the compressive strength test, samples with a thickness, length, and width of 1, 3, and 3 mm were cut from the cross-section of the specimen using a diamond saw and then dried and treated via high-pressure gold spraying. Subsequently, the samples were observed using a JSM-7500F SEM produced by Japan Electronics (Tokyo, Japan) with the best resolution of 1 nm (15 KV), and the micromorphological changes in the concrete after carbonation under freeze–thaw damage conditions were observed through scanning electron microscope experiments. To comprehensively understand the micropore structure information of concrete after the FTC and carbonation, samples that underwent 0, 100, and 200 FTCs and carbonation for 14 and 28 days were selected for nuclear magnetic resonance (NMR) analysis, and their porosity and PSD were tested. The test sample was processed into a cylinder with a diameter of 50 mm and a height of 50 mm using a drilling machine and a cutting machine to eliminate the influence of uneven freeze–thaw damage on the final experimental results relating to micropore structure. The porosity and PSD were tested using the MesoMR23-060H-I NMR analyzer produced by Suzhou Newman Analytical Instrument Co., Ltd., Suzhou, China. Before NMR testing, samples should be vacuumed and saturated. Thus, the specimen was placed into a vacuum water-retaining testing machine, the pumping time was set for 4 h, and the specimen was finally soaked in clean water for 24 h. To eliminate the influence of water evaporation on the test results, when the sample was taken out of the water, the water on the surface was wiped off, and the sample was wrapped in plastic and then placed on the NMR analyzer for measurement. Then, the porosity and PSD results could be obtained.

## 3. Experimental Results

### 3.1. Carbonization Depth

**Figure 3:**
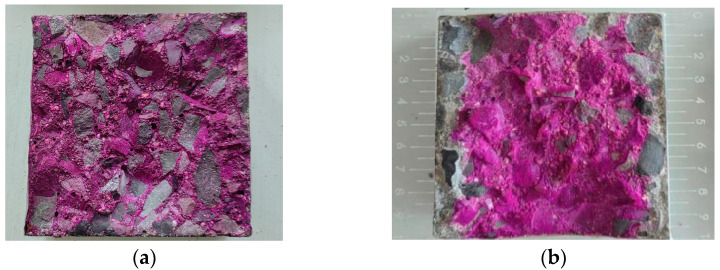
Measurement of carbonation depth of concrete (**a**) pre-carbonization specimen, (**b**) carbonized specimen.

The essence of carbonation reaction is acid–base neutralization reaction, which is a process in which the alkalinity of pore solution in concrete gradually decreases. The pH value of pore solution in uncarbonized concrete is generally about 13, and the greater the degree of carbonation, the lower the pH value. At present, the indicators for testing the carbonation depth of concrete mainly include phenolphthalein alcohol indicators and rainbow indicators [36,37]. In this study, the phenolphthalein indicator method was used to measure the carbonation depth of concrete. After the splitting test was completed, the specimen cross-section was cleaned with a soft brush to remove the residual powder, and a 1% concentration of phenolphthalein solution was sprayed on the splitting surface. In this test, the areas that have not undergone carbonization appear pink due to their alkalinity, while the areas that have undergone carbonization do not change color due to their neutrality. Then, after 30 s, a vernier caliper was used to measure the distance from the splitting surface to the junction of the discoloration area to obtain the depth of carbonization. The measurement of carbonization depth is shown in Figure 3. By measuring the depth of carbonation, the degree of concrete carbonation can be simply evaluated, and its compressive strength and durability can be determined. The carbonization depth of each point was measured on both sides (without discoloration) at marked intervals of 10 mm, and the average of multiple measurements was taken as the final carbonization depth.

The average carbonation depth of concrete at each test age can be calculated as follows:

(1) $$\bar{d_{i}}=\frac{1}{n}\overset{n}{\underset{i=1}{\sum }}d_{i},$$

where $\bar{d_{i}}$ is the average carbonization depth, mm; $d_{i}$ is the carbonization depth at measuring point *i,* mm; *n* is the total number of measurement points.

#### 3.1.1. Influence of FTCs on the Depth of Carbonization

**Figure 4:**
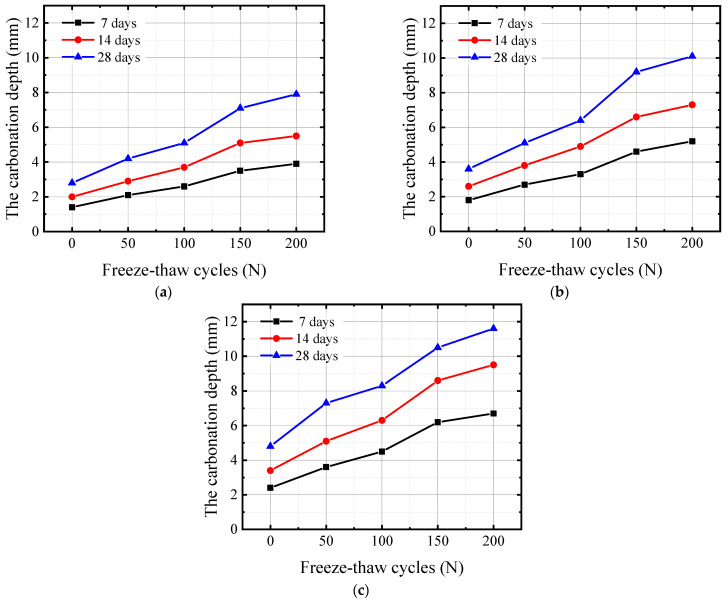
Relationship between the number of FTCs and the depth of carbonation for (**a**) 0.35 water–binder ratio, (**b**) 0.40 water–binder ratio, (**c**) 0.45 water–binder ratio.

The relationship between the carbonation depth and the number of FTCs for concrete specimens with 0.35, 0.40, and 0.45 water–binder ratios at 7, 14, and 28 days under FTC conditions was obtained on the basis of the color reaction of phenolphthalein as shown in Figure 4.

As shown in Figure 4, as the number of FTCs increases, the carbonization depth of all three groups of specimens increases. After 150 FTCs, the growth rate of the carbonation depth of the concrete decreases. During the FTC process, the pore water inside the concrete experiences volume expansion when it freezes; thus, the pores inside the concrete experience a certain degree of expansion and extension. After multiple FTCs, the porosity inside the concrete increases to a certain extent. The increases in porosity, microcracks, and connected pores all provide channels for CO_2_, which is conducive to its diffusion into the interior of concrete and promotes the carbonation reaction. Therefore, the carbonation depth increases with an increase in the number of FTCs.

After 150 FTCs, the carbonization reaction product CaCO_3_ adhering to the pore walls simultaneously envelops some Ca(OH)_2_, affecting the progress of the reaction, and blocks some pores in the concrete, affecting the diffusion rate of CO_2_ and thus affecting the progress of the carbonization reaction. Therefore, the growth rate of carbonization depth decreases in the later stage of FTCs.

#### 3.1.2. Relationship Between Carbonization Age and Carbonization Depth

**Figure 5:**
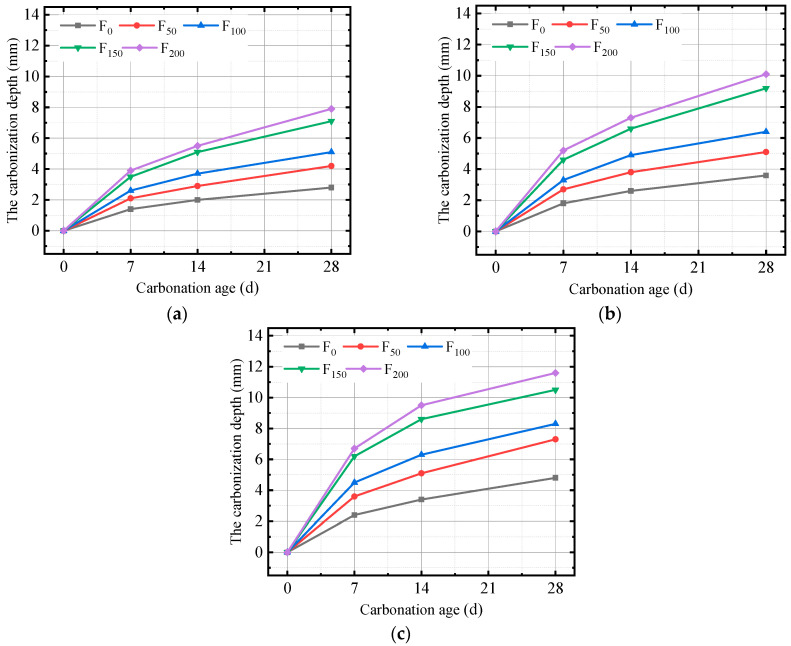
Relationship between carbonation age and the depth of carbonation for (**a**) 0.35 water–binder ratio, (**b**) 0.40 water–binder ratio, (**c**) 0.45 water–binder ratio.

As shown in Figure 5, the relationship between carbonization age and carbonization depth was plotted to investigate the influence of carbonization age on carbonization depth under freeze–thaw conditions.

Figure 5 shows that the carbonization depth of concrete increases with an increase in the number of FTCs and the carbonization age, and the carbonization depth of concrete after carbonization under the FTC condition is greater than that under the action of the carbonization test alone, which also indicates that the FTC can promote the carbonization reaction of concrete. With the extension of the carbonization age, the carbonization rate gradually decreases. The carbonation rate of the specimens with the three different water–binder ratios is faster than that of the concrete when carbonated for 7 days, and the carbonation rate gradually slows down after 14 days of carbonation.

In the early stage of carbonization, the FTC leads to an increase in porosity, and a relatively large CO_2_ concentration difference exists between the concrete interior and the surface layer, which provides more channels for the diffusion of CO_2_. In addition, the Ca(OH)_2_ content involved in the carbonization reaction is sufficient in the concrete, resulting in the acceleration of carbonization.

With the continuous progress of the reaction, the content of Ca(OH)_2_ in the solid phase is reduced, affecting the speed of the carbonization reaction. At the same time, the carbonization product CaCO_3_ continues to accumulate and fill the pores inside the concrete, increasing the compactness of the concrete and slowing down the diffusion rate of CO_2_ to the inside of the concrete, which contributes to the slowing down of the carbonization rate in the later stage.

#### 3.1.3. Effect of Water–Binder Ratio on Carbonization Depth

**Figure 6:**
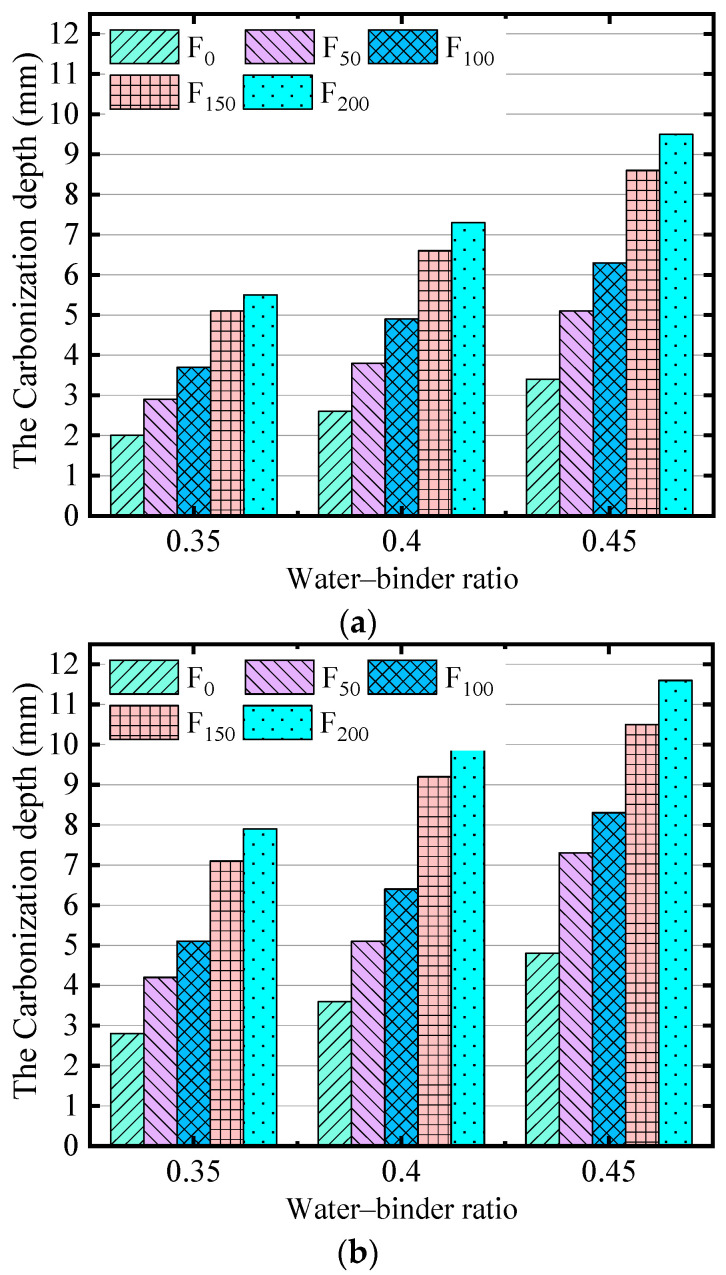
Relationship between water–binder ratio and the depth of carbonation for (**a**) 14 days of carbonation and (**b**) 28 days of carbonation.

The water–binder ratio has a great influence on the porosity of concrete, and the porosity connectivity directly affects the diffusion rate of CO_2_ in concrete. The relation between the water–binder ratio and carbonization depth of concrete at different ages under the action of FTCs is shown in Figure 6.

Figure 6 shows that, under the action of FTCs, the carbonization depth of the concrete increases with an increase in the water–binder ratio, and the greater the number of FTCs and the higher the water–binder ratio, the faster the growth rate of carbonization depth. The carbonization depths of the concrete with water–binder ratios of 0.35, 0.40, and 0.45 for 28 days were 2.8, 3.6, and 4.1 mm, respectively. After 200 FTCs, the 28-day carbonization depths were 7.8, 10.1, and 11.6 mm, increased by 1.79 times, 1.81 times, and 1.83 times, respectively. The larger the water–binder ratio, the more pores inside the concrete and the faster the carbonization reaction rate. By contrast, in the case of a certain unit of water consumption, when the water–binder ratio is increased, the amount of cementing material is reduced, the fluidity is increased, and more tiny pores and channels are formed after the evaporation of water during the hardening of the concrete. Therefore, increasing the water–binder ratio increases the porosity and pore connectivity of concrete and promotes the diffusion rate of H_2_O and CO_2_ in concrete, and the carbonization reaction is accelerated.

#### 3.1.4. Effect of Freeze–Thaw Damage on Carbonation Depth of Concrete

**Figure 7:**
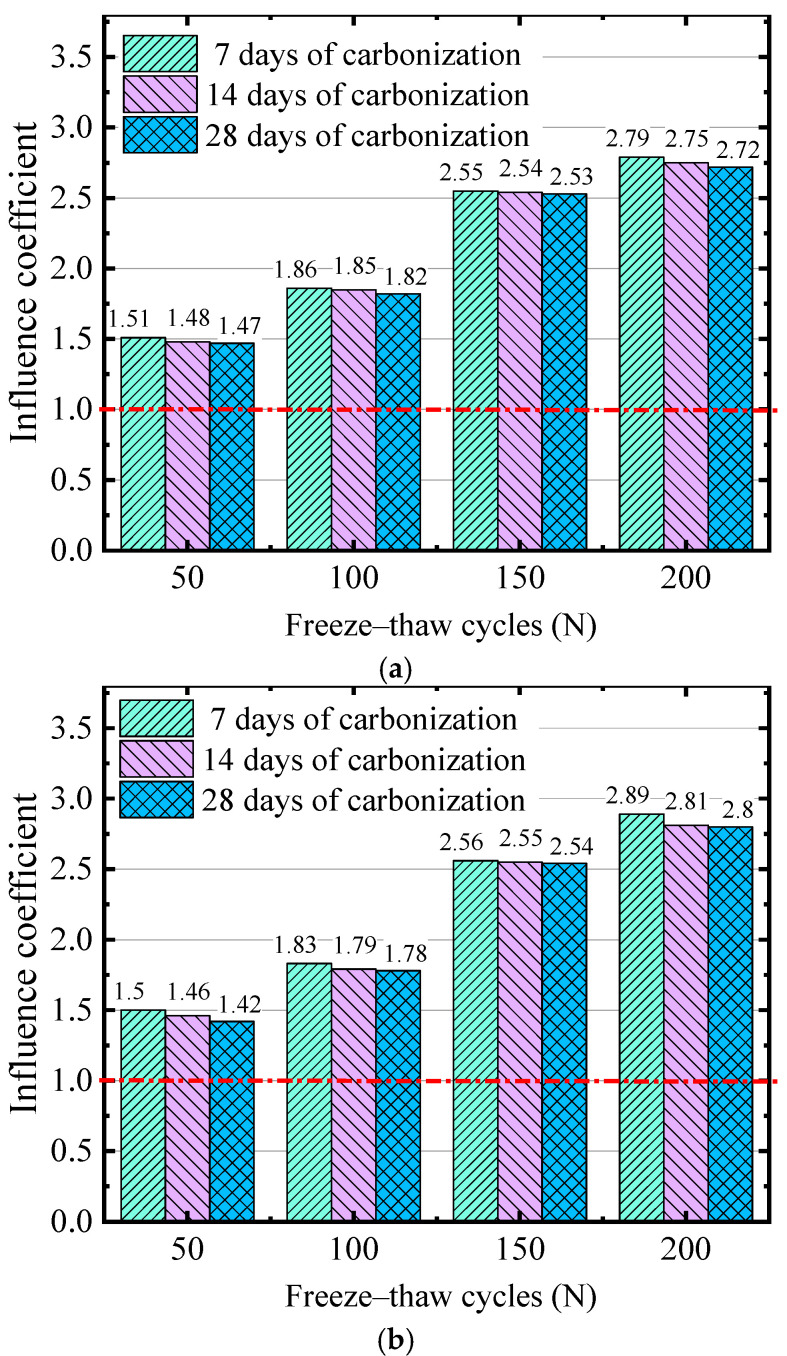

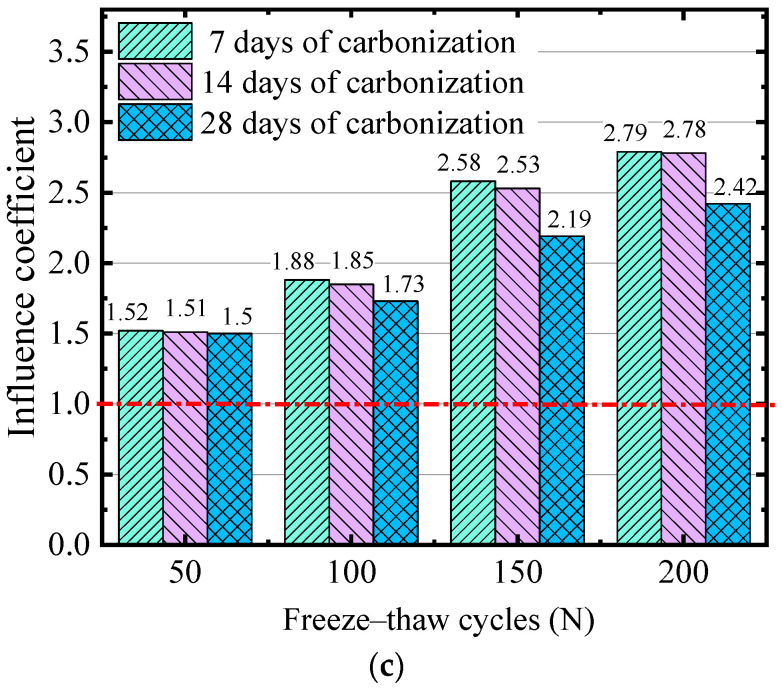
Relationship between the number of FTCs and influence coefficient of carbonization performance for (**a**) 0.35 water–binder ratio, (**b**) 0.40 water–binder ratio, (**c**) 0.45 water–binder ratio.

In combination with a single carbonation test and carbonation test data collected under freeze–thaw conditions, the influence of freeze–thaw damage on concrete carbonation performance is calculated and analyzed. The influence coefficient of the FTC on the carbonation performance of concrete is defined as $\lambda_{F}$ and is expressed as follows [38]:

(2) $$\lambda_{F}=d_{F+C}/d_{C},$$

where $d_{F+C}$ is the carbonization depth of the concrete after the FTC, $d_{C}$ is the carbonization depth of the concrete under carbonization, and $\lambda_{F}$ is the influence coefficient of the FTC on the carbonation depth of the concrete. $\lambda_{F}$ > 1 indicates that the FTC promotes carbonization, while $\lambda_{F}$ < 1 indicates that the FTC inhibits the development of carbonization depth to a certain extent. Figure 7 shows the influence coefficients of the three water–binder ratios on the carbonation performance of concrete under the action of FTCs.

Figure 7 shows that, after carbonization under freeze–thaw conditions, the influence coefficients of carbonization performance are all greater than 1 and increase with an increase in the number of FTCs. This result shows that the FTC has a promoting effect on carbonization, and the higher the number of FTCs, the more evident the promoting effect. With an increase in the number of cycles, the porosity inside the concrete may increase, and the connectivity between the pore size and pores also increases, providing convenient conditions for the diffusion of CO_2_ in the concrete and accelerating the occurrence of the carbonization reaction. The research of H. Kuosa [39] and Yang Li [33,40,41] confirmed that the carbonation depth of concrete increased with the increase in FTCs, which was attributed to the increase in surface cracking and internal microcracks of concrete caused by FTCs and the increase in porosity, which accelerated the carbonation reaction.

As shown in Figure 7a, 100 FTCs led to increases of 86%, 85%, and 82% in the carbonization depth after 7, 14, and 28 days of carbonization, respectively, and 200 FTCs led to increases of 179%, 175%, and 172% in the carbonization depth for the same carbonization durations. As shown in Figure 7b, 100 FTCs led to increases of 83%, 79%, and 78% in the carbonization depth after 7, 14, and 28 days, respectively, and 200 FTCs led to increases of 189%, 181%, and 180% in the carbonization depth for the same carbonization durations. As shown in Figure 7c, 100 FTCs led to increases of 88%, 85%, and 73% in the carbonization depth after 7, 14, and 28 days, respectively, while 200 FTCs led to increases of 179%, 178%, and 142% in the carbonization depth for the same carbonization durations. Therefore, under the same number of FTCs, the influence coefficient gradually decreases with an increase in carbonization age, which indicates that the influence of the freeze–thaw cycle on carbonization gradually weakens with an increase in carbonization age. With an increase in carbonization age, the products of the carbonization reaction fill part of the pores in the concrete, resulting in a decrease in porosity, which affects the carbonization reaction rate. Therefore, the carbonization reaction rate decreases in the later stage, and the influence of the FTCs on carbonization decreases.

### 3.2. Relative Dynamic Modulus of Elasticity

**Figure 8:**
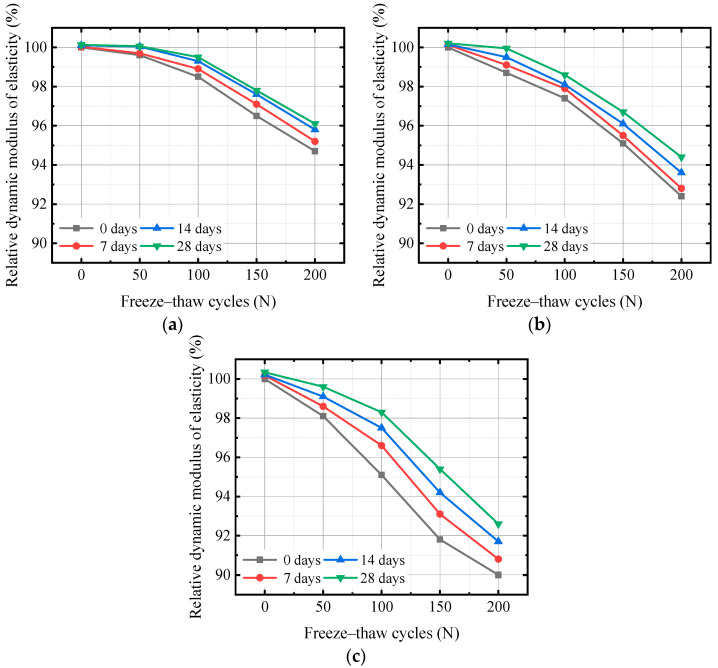
Relationship between the number of FTCs and the RDEM for (**a**) 0.35 water–binder ratio, (**b**) 0.40 water–binder ratio, (**c**) 0.45 water–binder ratio.

The changes in the RDEM of concrete with different water–cement ratios after carbonation under FTC conditions are shown in Figure 8.

Figure 8 shows that the RDEM of concrete after carbonation under freeze–thaw conditions decreases with an increase in the number of FTCs. As the water–cement ratio increases, the RDEM of concrete continuously decreases, and the larger the water–cement ratio, the larger the decrease. For unfrozen specimens, the RDEM after carbonization is greater than 100%. After 200 FTCs, the RDEMs of the concrete specimens with 0.35, 0.40, and 0.45 water–cement ratios without carbonation were 94.7%, 92.4%, and 90%, respectively. After 28 days of carbonation, they were 96.1%, 94.4%, and 92.6%, respectively. The carbonation effect at 28 days resulted in increases of 1.48%, 2.16%, and 2.89% in the RDEM. These results indicate that carbonation improves the RDEM of concrete, restrains the expansion of freeze–thaw degradation characteristics, and alleviates the damage caused by FTCs to concrete. This conclusion is consistent with the research results in the literature.

Under the action of FTCs, the internal structure of concrete gradually transforms from dense to porous, and microcracks gradually expand and extend, leading to an increase in porosity. In a rapid carbonation environment, CO_2_ with strong permeability enters the interior of concrete through pores, undergoes a neutralization reaction with the Ca(OH)_2_ in the pores, and alleviates the damage to the RDEM under FTCs.

### 3.3. Compressive Strength

**Figure 9:**
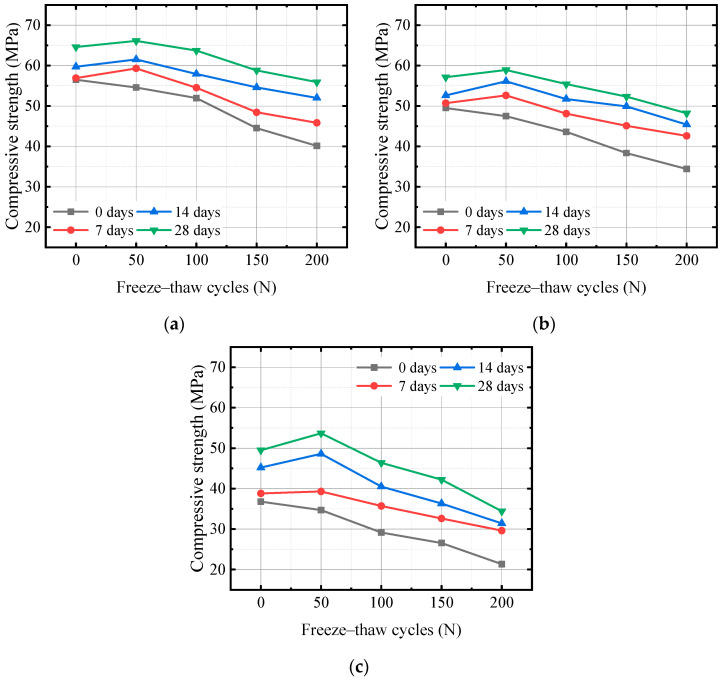
Relationship between carbonization age and compressive strength for (**a**) 0.35 water–binder ratio, (**b**) 0.40 water–binder ratio, (**c**) 0.45 water–binder ratio.

The compressive strength of concrete with three water–binder ratios after carbonation under freeze–thaw damage conditions is shown in Figure 9. As shown in the figure, as the carbonization age increases, the compressive strength of concrete increases, and the higher the water–cement ratio, the faster the growth rate of compressive strength after carbonization. For the group of specimens that underwent 0 FTCs, after 28 days of carbonization, the compressive strength of the specimens with 0.35, 0.40, and 0.45 water–binder ratios increased from 56.5, 49.4, and 36.8 MPa to 64.6, 57.1, and 49.5 MPa, respectively, and increased to 114.34%, 115.35%, and 134.51% of their respective compressive strength before carbonization. Thus, the compressive strength of concrete shows a trend of first increasing fast and then increasing slowly with an increase in carbonization age. After 14 days of carbonization, the increase in strength was relatively slow. During the carbonation reaction of concrete, calcium hydroxide reacts with CO_2_ to generate CaCO_3_, which increases the volume of the generated product relative to the reactant and plays a particular filling role in the pore structure, making the pore structure denser and improving the strength of the concrete. The diffusion rate of CO_2_ in concrete directly affects the rate of the carbonation reaction.

Combining the subfigures of Figure 9 reveals that carbonization can alleviate the damage to concrete strength caused by FTCs. The increase in compressive strength after 7 days of carbonization was greater than the damage to the compressive strength caused by 50 FTCs. After 100 FTCs, the carbonization reaction was insufficient to compensate for the strength loss caused by freeze–thaw damage. For the specimens that underwent 200 FTCs, the 28 days of carbonization resulted in a 39.33% increase in the compressive strength of the 0.35 water–binder ratio concrete compared with the concrete, a 40.12% increase in the compressive strength of the 0.40 water–binder ratio concrete, and a 61.5% increase in the compressive strength of the 0.45 water–binder ratio concrete. Concrete specimens with a large water–binder ratio have a large internal porosity and relatively loose pore structure, resulting in a higher diffusion rate of carbon dioxide, which leads to a faster carbonation reaction rate and a larger content of calcium carbonate generated. The rate of filling dense pores with the generated product is also relatively faster, resulting in an accelerated rate of strength improvement. Therefore, the higher the water–binder ratio of concrete, the faster the strength growth rate after carbonization. Reference [4] also confirmed that the water–binder ratio is the main reason that affects the carbonation performance of concrete, and carbonation improves the compactness and mechanical properties of concrete under FTCs.

Carbonization products fill some pores, which blocks the pores’ connectivity. The diffusion of CO_2_ is blocked, and the carbonization reaction speed slows down, which leads to slow strength growth after 14 days of carbonization. After 50 FTCs, the damage to the compressive strength was relatively small, and the increase in concrete strength caused by carbonation was greater than the damage caused by FTC. After 100 FTCs, the damage to the concrete caused by FTCs was evident, and the increase in the compressive strength caused by the carbonation reaction was not enough to compensate for the damage to the concrete compressive strength.

### 3.4. Microstructure Evolutions

#### 3.4.1. Micromorphology

**Figure 10:**
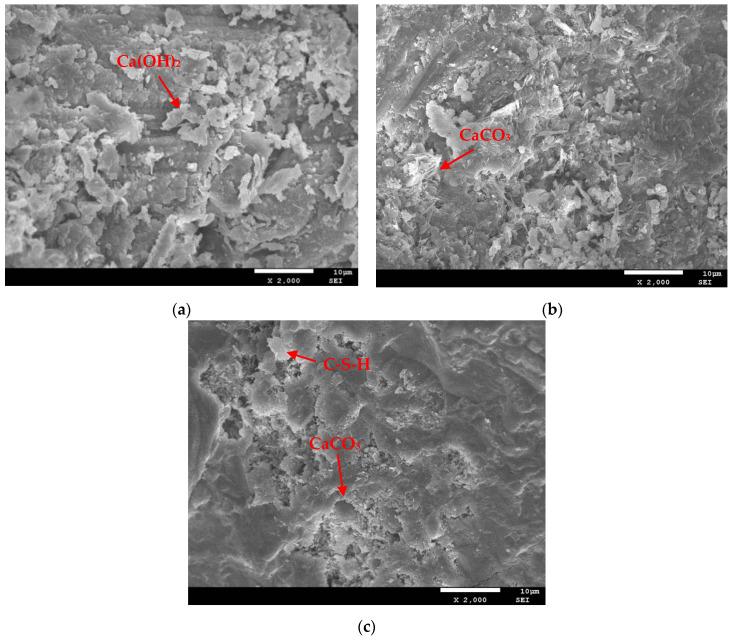
Electron microscope scanning of concrete after carbonization: (**a**) carbonization for 0 days, (**b**) carbonization for 14 days, (**c**) carbonization for 28 days.

SEM images of concrete samples with different carbonization ages at a magnification of 2000 are shown in Figure 10.

As shown in Figure 10c, the number of pores further decreases, indicating that the concrete becomes denser after carbonation. A comprehensive comparison of the micro changes in the surface of concrete reveals that, with an increase in the number of carbonation days, the pore structure of concrete undergoes considerable changes; the smoothness of the concrete surface is enhanced, the integrity is increased, and the number of small pores is reduced. The reason for this is that, during carbonation, the Ca(OH)_2_ in the concrete reacts with CO_2_ and the carbonization product CaCO_3_, which fills the interior of the concrete, thereby reducing the porosity of the internal structure of the concrete, increasing the compactness of the concrete, and improving its strength.

**Figure 11:**
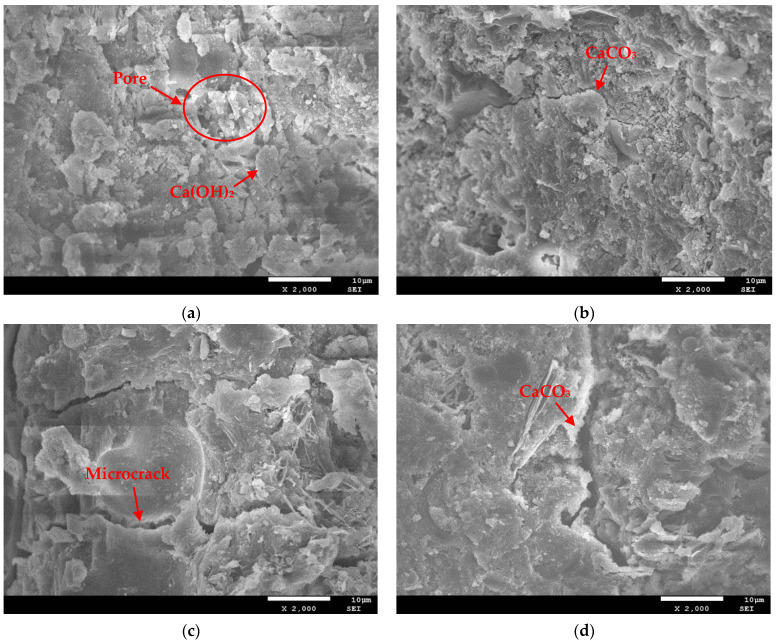
Electron microscope scanning of concrete after carbonization under freeze–thaw conditions: (**a**) carbonization for 0 days under 100 FTCs, (**b**) carbonization for 28 days under 100 FTCs, (**c**) carbonization for 0 days under 200 FTCs, (**d**) carbonization for 28 days under 200 FTCs.

SEM analysis was conducted on concrete specimens that underwent 100 FTCs, 200 FTCs, and carbonation for 0 and 28 days. The microstructure of the samples at a magnification of 2000 is shown in Figure 11.

Figure 11a shows that the concrete that underwent 100 FTCs has spherical fly ash particles without complete hydration on its surface before carbonization. Under the freeze–thaw action, cracks appear, exhibiting more pores, and the surface is rugged with many thin-layered and spherical Ca(OH)_2_ crystals. Figure 11b shows a sample that underwent 100 FTCs and 28 days of carbonization. The surface still shows many cracks and pores, but compared with Figure 11a, the hydration products on the surface are considerably reduced, the width of the cracks is narrowed, and the structure is complete and dense. During the carbonization reaction, CO_2_ reacts with Ca(OH)_2_ to generate CaCO_3_, which consumes a portion of Ca(OH)_2_ and flattens the structure. The reaction products fill some pores and cracks, and various hydration products connect to form a whole, making the structure dense, reducing the porosity of the concrete, and improving its strength. Figure 11c shows that, after 200 FTCs, microcracks further increase, the aggregate detaches from the slurry, gaps are visible at the cross-section, and the surface becomes loose and porous. As shown in Figure 11d, carbonization causes the structure to become more complete and denser than that under 200 FTCs with 0 days of carbonization, and the carbonization reactant CaCO_3_ can be seen attached to the pore wall at the edge of the crack. However, freeze–thaw damage remains relatively evident, and the carbonization products are not enough to fill the cracks caused by FTCs. This result explains the decrease in the RDEM, ultrasonic wave velocity, splitting tensile strength, and compressive strength from a microscopic perspective.

#### 3.4.2. Pore Structure

##### Porosity

**Figure 12:**
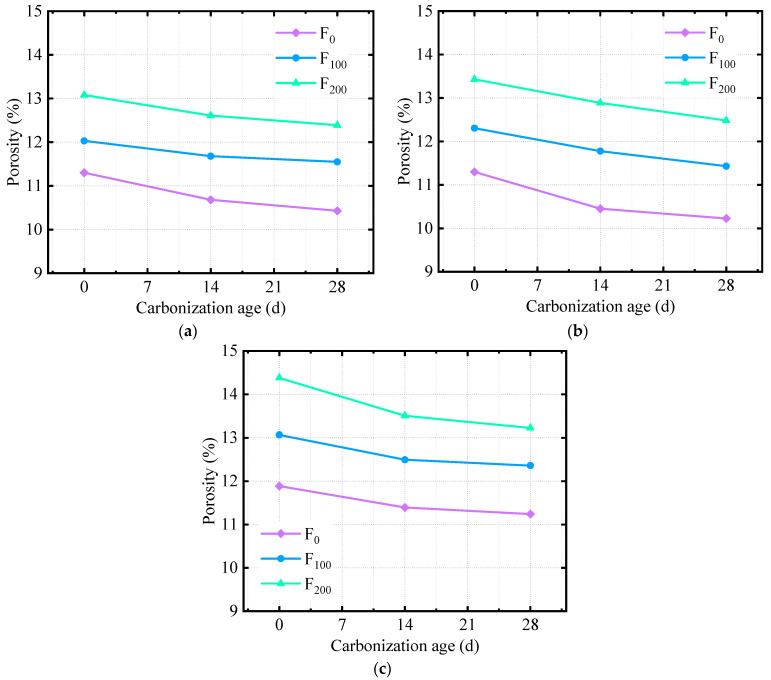
Porosity after carbonation under freeze–thaw conditions for (**a**) 0.35 water–binder ratio, (**b**) 0.40 water–binder ratio, (**c**) 0.45 water–binder ratio.

In order to investigate the influence mechanism of FTCs on the carbonation of concrete, three water–binder ratios of concrete were subjected to 0, 100, and 200 FTCs and then placed in a carbonation box to undergo accelerated carbonation for 14 and 28 days. Finally, NMR analysis was performed on the samples to obtain their porosity. The relationship between the carbonation age and porosity of concrete under FTCs is shown in Figure 12.

Figure 12 shows that, under freeze–thaw damage conditions, the porosity of the carbonated concrete increases with an increase in the number of FTCs and the water–cement ratio. As the carbonization age increases, the porosity gradually decreases, and after 14 days of carbonization, the rate of porosity change slows down. Freeze–thaw damage leads to an increase in the porosity of concrete, affecting the diffusion of CO_2_ inside the concrete and accelerating the carbonation process. The higher the number of FTCs, the greater the acceleration effect. As the water–binder ratio increases, more small pores and channels are formed by water evaporation after concrete hardening. The internal porosity of the concrete is relatively high, and the structure is relatively loose, promoting the diffusion of water and CO_2_ in the concrete, thereby accelerating the rate of carbonation reaction and generating more CaCO_3_. As the carbonization age increases, CO_2_ reacts with Ca(OH)_2_ to form CaCO_3_, which has a particular filling effect on the pore structure and reduces the porosity. However, after a carbonization reaction period (14 days), as the internal pores become denser, the generated carbonization product CaCO_3_ fills some of the pores, playing a blocking role, which hinders the diffusion process of CO_2_ and reduces the rate of porosity change.

##### Pore Size Distribution

**Figure 13:**
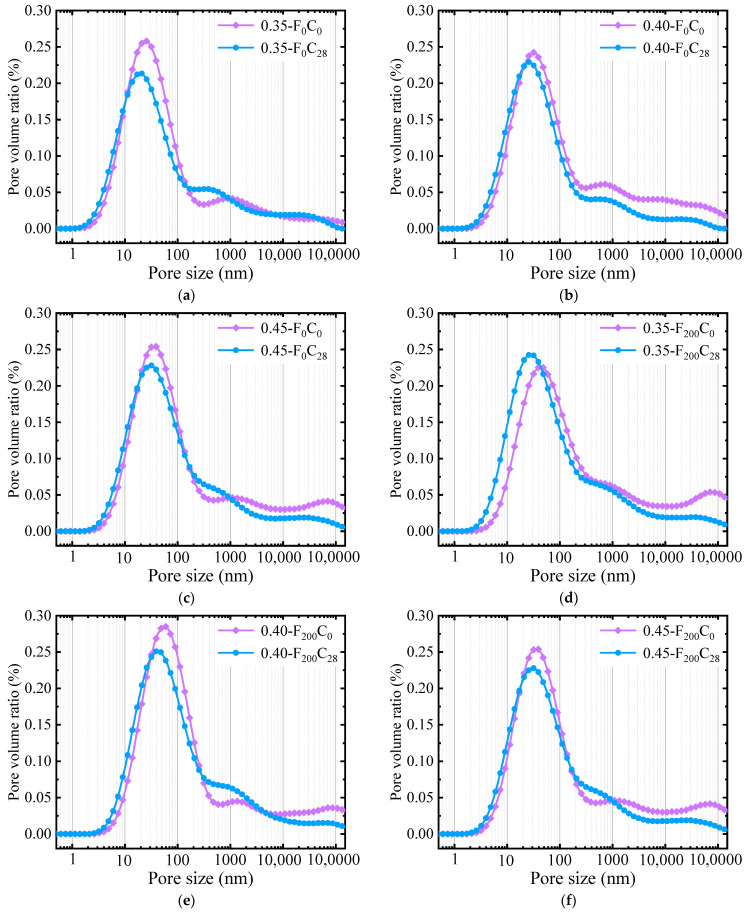
PSD before and after carbonization under FTC conditions for (**a**) 0.35 water–binder ratio, 0 FTCs, (**b**) 0.40 water–binder ratio, 0 FTCs, (**c**) 0.40 water–binder ratio, 0 FTCs, (**d**) 0.35 water–binder ratio, 200 FTCs, (**e**) 0.40 water–binder ratio, 200 FTCs, (**f**) 0.40 water–binder ratio, 200 FTCs.

The porosity, PSD, and pore connectivity of concrete directly affect the transport characteristics and mechanical properties of water, gas, and ions in concrete. As PSD can better evaluate the comprehensive performance of hydrated concrete, the concrete specimens that underwent 0 and 28 days of carbonization after 0 and 200 FTCs were selected as samples. A magnetic resonance analyzer was used to analyze the influence of carbonization on the PSD of concrete under FTC damage conditions, as shown in Figure 13.

Figure 13 shows that carbonization changes the PSD of the concrete. Whether it occurs before or after the FTC, the average pore diameter and the most available pore diameter decrease, and the total porosity decreases. Figure 13a–c shows that, for noncarbonized specimens that underwent 0 FTCs, the most probable pore sizes for the concrete with 0.35, 0.40, and 0.45 water–binder ratios were 24.07, 31.77, and 36.50 nm, respectively. After 28 days of carbonization, the most probable pore sizes decreased to 20.45, 25.8, and 29.48 nm, respectively, decreases of 15.04%, 18.79%, and 19.23%. The greater the water–binder ratio, the more the most probable pore size decreased. Figure 13d–f shows that, after 200 FTCs and before carbonization, the maximum pore sizes of the concrete with the three water–binder ratios before carbonization were 41.94, 59.35, and 73.09 nm, respectively, and the maximum pore sizes were reduced to 21.44, 41.94, and 44.96 nm after 28 days of carbonization—decreases of 25.04%, 29.33%, and 38.49%. Carbonization reduces the most probable pore size of concrete before and after the FTC, and the greater the water–binder ratio, the greater the change value. The carbonization reaction produces CaCO_3_, which adheres to the pore wall, resulting in a decrease in the pore diameter participating in the reaction, changing the pore diameter distribution, and reducing the most probable pore size of the concrete. The larger the water–binder ratio, the larger the initial porosity, and the greater the damage under the FTCs, which speeds up the diffusion rate of CO_2_ in concrete and accelerates the carbonization reaction rate. Therefore, the larger the water–binder ratio, the faster the reduction rate of the most probable pore size.

**Figure 14:**
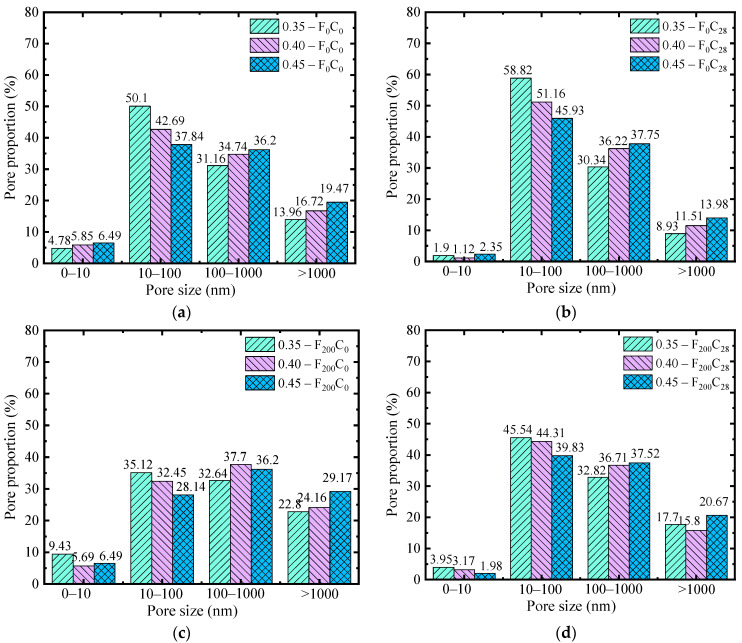
PSD of specimens with different corrosion rates: (**a**) 0 FTCs, carbonization for 0 days, (**b**) 0 FTCs, carbonization for 28 days, (**c**) 200 FTCs, carbonization for 0 days, (**d**) 200 FTCs, carbonization for 28 days.

To further explore the influence mechanism of the micropore structure of carbonized concrete on macroproperties under FTC conditions, on the basis of the results of NMR analysis and referencing the literature [42,43] on pore structure classification, the pore structure of carbonized concrete under freeze–thaw conditions was classified, and its PSD was statistically analyzed. Different types of pore distribution are plotted in Figure 14.

Figure 14 shows that, after 28 days of carbonization, the gel pores (d < 10 nm), transition pores (10 nm ≤ d < 100 nm), and macropores (d > 1000 nm) changed considerably. The content of gel pores and macropores decreased, and the content of transition pores increased. For capillary pores (100 nm ≤ d < 1000 nm), a slight fluctuation occurred, but the change was not considerable. The carbonization reaction affects all four kinds of pores, but the evolution law of pores states that the reaction degree is different. CO_2_ gradually diffuses from the surface to the interior of the concrete pores under the effect of concentration difference and reacts with Ca(OH)_2_ in the pores to generate CaCO_3_, which is attached to the pore wall, resulting in a smaller pore size and lower porosity. The pore diameter of the gel becomes smaller and cannot be detected by the NMR analyzer; thus, the gel pore content decreases. The considerable decrease in macropore content is due to the large surface area of macropores, which generate more CaCO_3_ in full contact with CO_2_, resulting in a considerable reduction in pore size. The increase in transitional pore content is due to the fact that, although carbonization occurs in the range of 10 nm ≤ d < 100 nm, the carbonization reaction speed is faster in pore sizes of 100 nm ≤ d < 1000 nm and d > 1000 nm, and the pore size decreases more, ultimately leading to an increase in pore size content in the range of 10 nm ≤ d < 100 nm.

Figure 14a,c show that, under the action of the FTC, the percentage of transition pores with a pore size of 10 nm ≤ d < 100 nm decreases, the percentage of macropores with d > 1000 nm increases, and the amplitude of gel pores with d < 10 nm and capillary pores with a pore size of 100 nm ≤ d < 1000 nm is not evident, indicating that the FTC has no considerable effect on gel pores. The FTC causes the change in pore size when d ≤ 100 nm, which ultimately increases macropores and decreases transition pores.

Combining the results of the FTC and carbonization testing, including RDEM and compressive strength values, reveals that the reason for the increase in porosity and strength after carbonization is the decrease in macropore content with d > 1000 nm and the increase in transitional pore content with a pore size of 10 nm ≤ d < 100 nm caused by carbonization. Controlling the content of large pores with d > 1000 nm and increasing the content of transition pores with a pore size of 10 nm ≤ d < 100 nm can improve the carbonation durability of concrete under FTC conditions.

## 4. Discussion

On the basis of the gray relational analysis model, the influence of pore structure on the RDEM, carbonation depth, and compressive strength of concrete under the combined action of FTCs and carbonation is discussed. Through fitting analysis between porosity and carbonation depth, the relationship between pore structure and carbonation depth is obtained, and the influence mechanism of the FTC on carbonation depth is discussed from macro to micro perspectives.

### 4.1. Analysis of Influencing Factors Based on Gray Correlation Model

The gray correlation analysis method measures the correlation program between factors based on their development trend. The degree of correlation directly reflects the degree of influence of various factors in the system on the target value, indicating the degree of correlation between two systems or factors [44,45]. By calculating the correlation between the target value (reference sequence) and the influencing factors (comparison series), the correlation is sorted to identify the main factors that affect the target value. By conducting data calculations on the various factors that must be analyzed, the correlation can be found in a random sequence of factors, and the main influencing factors can be identified. The general calculation steps for factor analysis using the gray correlation degree are as follows:

(1) Establish reference and comparison series.

$X_{0}(k)=\left\{X_{0}\left(1\right),X_{0}\left(2\right),X_{0}\left(3\right),\cdots ,X_{0}\left(n\right)\right\}$ is set as the system reference series and $X_{i}(k)=\left\{X_{i}\left(1\right),X_{i}\left(2\right),X_{i}\left(3\right),\cdots ,X_{i}\left(m\right)\right\}$ as the comparison sequence of m-many related factors in the model. Six indicators reflecting the durability of concrete against carbonation erosion, i.e., the RDEM, carbonation depth, porosity, compressive strength, tensile compressive strength ratio, and carbonation performance influence coefficient, are used as reference sequences for the model. Three factors that affect carbonization durability, i.e., the water–binder ratio, FTCs, and carbonization age, are set as comparative series.

(2) Initialize each sequence as follows:

(3) $$X_{j}(k)=\frac{X_{j}\left(k\right)}{\max_{k}X_{j}\left(k\right)},$$

where j=0, 1, 2, ⋯, m.

(3) Calculate the correlation coefficient.

The correlation between the comparison sequence and the reference sequence is as follows:

(4) $$\xi_{i}(k)=\frac{\min_{i}\min_{k}\left|X_{0}\left(k\right)-X_{i}\left(k\right)\right|+\rho \max_{i}\max_{k}\left|X_{0}\left(k\right)-X_{i}\left(k\right)\right|}{\left|X_{0}\left(k\right)-X_{i}\left(k\right)\right|+\rho \max_{i}\max_{k}\left|X_{0}\left(k\right)-X_{i}\left(k\right)\right|}$$

In the formula, $\xi_{i}(k)$ is the correlation coefficient between element k of comparison sequence $X_{i}$ and element k of reference sequence $X_{0}$; ρ is the resolution coefficient, where $\rho \in \left[0,1\right]$, usually taken as 0.5.

(4) Solve the correlation degree.

The correlation degree is calculated according to the following equation:

(5) $$\gamma_{i}=\frac{1}{n}\overset{n}{\underset{k=1}{\sum }}\xi_{i}\left(k\right)$$

In the equation, $\gamma_{i}$ is the correlation between the comparison sequence $X_{i}$ and the reference sequence $X_{0}$.

(5) Sort association order.

On the basis of the degree of correlation, the order of the impact of each factor on the target value is determined. The magnitude of the correlation degree determines the importance of the influencing factors; the larger the correlation degree value, the closer the development trend of $X_{i}$ and $X_{0}$ is, and the greater the effect of $X_{i}$ on $X_{0}$.

#### 4.1.1. Correlation Analysis of Macro-Influencing Factors

Five durability indexes of carbonation resistance of reactive concrete, such as RDEM, carbonation depth, porosity, compressive strength, and carbonation performance influence coefficient, are taken as the model reference sequence. The water–binder ratio, FTCs, carbonation age, and three other factors that affect carbonation durability are set as a comparison series. According to the calculation method of grey correlation degree, the macro-influencing factors are analyzed by grey correlation degree, and the results are shown in Table 2.

As shown in Table 2, the influence of water–binder ratio, freeze–thaw cycles, and carbonation age on the RDEM and porosity of concrete after carbonation under freeze–thaw conditions is as follows: water–binder ratio > FTCs > carbonation age, and the water–binder ratio has the greatest influence. The correlation coefficients of the water–binder ratio to RDEM and porosity are 0.879 and 0.855, which show a strong correlation. The order of influence of different factors on carbonation depth is carbonation age (0.774) > FTCs (0.659) > water–binder ratio (0.656). For the compressive strength, the influence of different factors is as follows: water–binder ratio (0.783) > carbonation age (0.61) > FTCs (0.568). The water–binder ratio and the number of FTCs have the greatest influence on the carbonation performance, followed by carbonation age. Thus, under the combined action of FTC and carbonation, the carbonation age has the greatest influence on carbonation depth, and the water–binder ratio has the greatest influence on other macro properties of concrete, while the carbonation age and FTCs have relatively little influence. Properly increasing the water–binder ratio can improve the carbonation resistance of concrete under freeze–thaw conditions.

#### 4.1.2. Correlation Analysis of Influencing Factors of Pore Structure

According to the calculation method of the gray correlation degree, the RDEM, carbonization depth, and compressive strength are taken as the reference sequence of the model, and the gel hole (d < 10 nm), transition hole (10 nm ≤ d ≤ 100 nm), capillary hole (100 nm ≤ d ≤ 1000 nm), and large hole (d > 1000 nm) are set as the comparison series for gray correlation degree analysis. Table 3 shows the calculation results of the correlation degree coefficients.

As shown in Table 3, the capillary pores (100 nm ≤ d ≤ 1000 nm) and transition pores (10 nm ≤ d ≤ 100 nm) have a great influence on the carbonization depth under freeze–thaw conditions, and the correlation coefficients are 0.653 and 0.643. The order of influence on the RDEM is capillary hole > transition hole > macropore > gel hole. The capillary hole has the greatest influence, and the correlation coefficient exceeds 0.8, showing a strong correlation. The order of influence on compressive strength is transition pore > capillary pore > macropore > gel pore, and the correlation coefficients are 0.907, 0.749, 0.607, and 0.547, respectively. After carbonization under freeze–thaw conditions, capillary pores (100 nm ≤ d ≤ 1000 nm) have the greatest influence on the RDEM and carbonization depth, followed by transition pores (10 nm ≤ d ≤ 100 nm), large pores (d > 1000 nm), and gel pores (d < 10 nm). Under freeze–thaw conditions, the effect of gel pores on the macroscopic properties of concrete is small. Reducing the content of large pores and increasing the content of transition pores and capillary pores can improve the compressive strength, RDEM, and carbonization properties of concrete.

### 4.2. Relationship Between Carbonization Depth and Pore Structure

**Figure 15:**
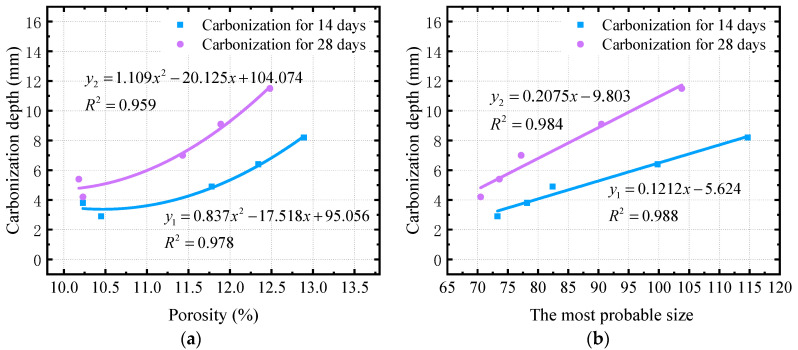
Relationship between carbonation depth and (**a**) porosity, (**b**) the most probable size.

The relationship between the carbonation depth, porosity, and most probable pore size of concrete after carbonation under freeze–thaw conditions is shown in Figure 15. The relationship between the carbonation depth and porosity of the concrete demonstrates a nonlinear positive correlation after carbonation for 14 days and 28 days, with correlation coefficients of 0.978 and 0.959, respectively. The relationship between the carbonation depth and the most probable pore size reveals a linear positive correlation, with correlation coefficients of 0.988 and 0.984, respectively, showing excellent correlations.

### 4.3. Effect Mechanism of Freeze–Thaw Cycle on Carbonation

The carbonation mechanism of concrete under freeze–thaw damage conditions is basically the same as that in general atmospheric environments. At the early stage of the FTC, microcracks occur in volume-expanded concrete due to freezing. With an increase in the number of FTCs, the internal microcracks gradually develop, the mortar layer on the surface of the concrete spalls, and the structure becomes loose and porous. The porosity inside the concrete also changes greatly, and the number and pore size of the pores increase, which is conducive to the diffusion of CO_2_ and speeds up the carbonization reaction. As the carbonization reaction continues, CO_2_ and Ca(OH)_2_ inside the concrete react chemically, resulting in CaCO_3_ attached to the inner pore wall of the concrete, which fills the pores inside the concrete to a certain extent, resulting in a decrease in the porosity inside the concrete, an increase in the compactness of the concrete, and an increase in strength. With the later process of carbonization, the pore structure of concrete is blocked by the generated CaCO_3_, resulting in an increase in the internal compactness of the concrete and a decrease in pore volume, which is the reason for the decrease in porosity. At the same time, this process prevents external CO_2_ from entering the concrete pores and undergoing the carbonizing reaction. Thus, after 14 days of carbonization, the strength increase is relatively slow, which is the reason for the low carbonization reaction rate in the later stage.

The FTC accelerates the carbonation reaction and reduces the durability of concrete, which is also confirmed by the research of TIAN Li [46] and NIU Ditao [38]. When freeze–thaw and carbonation phenomena are observed in the actual service environment, predicting service life and design durability based only on the carbonation law determined by a single carbonation factor in the laboratory is unreasonable. The combined effects of the FTC, carbonation, and other factors must be considered.

## 5. Conclusions

In this study, freeze–thaw cycle and carbonation experiments were conducted, and the relationship between carbonation depth and the microstructure of concrete mixed with fly ash under FTCs was analyzed. The results of this study are summarized in the concluding remarks below.

(1) The freeze–thaw cycle, as the power source of concrete damage, increases the porosity of concrete, which creates favorable conditions for the carbonation reaction and thus accelerates the process. Carbonization improves the compressive strength of concrete under freeze–thaw damage. The compressive strength of concrete specimens with water–binder ratios of 0.35, 0.40, and 0.45 increased by 39.3%, 40.2%, and 61.4%, respectively, after 200 FTCs.
(2) The carbonation depth of concrete increases with the number of FTCs and the water–binder ratio. Carbonization improves the RDEM of concrete, inhibits the expansion of freeze–thaw deterioration characteristics, and alleviates the damage to the concrete caused by the FTC. Carbonization changes the microstructure of concrete, reducing the porosity and the most probable pore size of concrete under freeze–thaw damage conditions.
(3) The content of macropores with d > 1000 nm decreases, and the content of transition pores with a pore size of 10 nm ≤ d < 100 nm increases after carbonation under freeze–thaw damage conditions, which is the direct reason for the decrease in porosity and the improvement in strength. Therefore, controlling the content of macropores with d > 1000 nm and increasing the content of transition pores with a pore size of 10 nm ≤ d < 100 nm can improve the carbonation durability of fly ash concrete under freeze–thaw conditions.
(4) Gray relational analysis showed that the carbonation age has the greatest influence on carbonation depth, and the water–binder ratio has the greatest influence on other macroproperties of concrete. Thus, properly reducing the water–binder ratio can improve the carbonation resistance of concrete under freeze–thaw conditions. The capillary hole (100 nm ≤ d ≤ 1000 nm) has the greatest influence on the RDEM and carbonation depth, followed by the transition hole (10 nm ≤ d ≤ 100 nm), and then the macropore (d > 1000 nm), and the gel hole (d < 10 nm) has the least influence.
(5) The relationship between the carbonation depth and pore structure of concrete shows a nonlinear positive correlation between carbonation depth and porosity under freeze–thaw conditions and a linear positive correlation with the most probable pore size.

## Figures and Tables

**Table 1:** Design of mix proportion of the concrete specimens (in kilograms per cubic meter).

Water–Cement Ratio	Cement	Fly Ash	Sand	Stone	Water	Water-Reducing Agent	Air-Entraining Agent
0.35	295	74	711	1202	129	1%	0.2%
0.40	258	65	728	1240	129	1%	0.2%
0.45	229	57	742	1264	129	1%	0.2%

**Table 2:** Calculation results of grey correlation degree of macro-influencing factors.

Influencing Factor	RDEM	Carbonization Depth	Porosity	Compressive Strength	Influence Coefficient of Carbonization Performance
water–binder ratio	0.879	0.656	0.885	0.783	0.702
FTCs	0.579	0.659	0.576	0.568	0.704
carbonation age	0.547	0.774	0.550	0.610	0.588

**Table 3:** Calculation results of correlation degree coefficient of pore structure parameters.

Pore Diameter	RDEM	Carbonization Depth	Compressive Strength
Gel pore (d < 10 nm)	0.597	0.539	0.547
Transition pore (10 nm ≤ d ≤ 100 nm)	0.818	0.643	0.907
Capillary pore (100 nm ≤ d ≤ 1000 nm)	0.890	0.653	0.749
Macro pore (d > 1000 nm)	0.714	0.595	0.607

## Data Availability

The original contributions presented in this study are included in the article. Further inquiries can be directed to the corresponding author.

## References

[1] Quan H.Z. (2012). Research on durability of high volume fly ash concrete. Appl. Mech. Mater..

[2] Wang R., Zhang Q., Li Y. (2022). Deterioration of concrete under the coupling effects of freeze–thaw cycles and other actions: A review. Constr. Build. Mater..

[3] Li J., Yang H., Wu H. (2024). Evaluation of Concrete Carbonation Based on a Fiber Bragg Grating Sensor. Micromachines.

[4] Yang Q., Liu M., Li J., Chen X. (2024). Study on the degradation models based on the experiments considering the coupling effect of freeze-thaw and carbonation. Structures.

[5] Zhang D., Yang Q., Mao M., Li J. (2020). Carbonation performance of concrete with fly ash as fine aggregate after stress damage and high temperature exposure. Constr. Build. Mater..

[6] Dhanesh S., Kumar K.S., Maruthur P., Rejumon R., Usmansha G.S. (2021). Experimental investigation of strength of Aramid kelvar and chopped carbon reinforced concrete beam. Mater. Today: Proc..

[7] Wang X., Yang Q., Peng X., Qin F. (2024). A Review of Concrete Carbonation Depth Evaluation Models. Coatings.

[8] Sun X., Cui Y., Chen J., Yi S., Li X., Chen L. (2024). MFRWA: A Multi-Frequency Rayleigh Wave Approximation Method for Concrete Carbonation Depth Evaluation. Buildings.

[9] Moghaddas S.A., Nekoei M., Golafshani E.M., Nehdi M., Arashpour M. (2022). Modeling carbonation depth of recycled aggregate concrete using novel automatic regression technique. J. Clean. Prod..

[10] Qu F., Xia W., Sun C., Hou H., Huang B., Wang G., Hu S. (2024). Modeling carbonation depth of recycled aggregate concrete containing chlorinated salts. Constr. Build. Mater..

[11] Xiao Q.H., Li Q., Guan X., Zou Y.X. (2018). Prediction model for carbonation depth of concrete subjected to freezing-thawing cycles. IOP Conf. Ser. Mater. Sci. Eng..

[12] You Q., Ren G., Fraedrich K., Kang S., Ren Y., Wang P. (2013). Winter temperature extremes in China and their possible causes. Int. J. Clim..

[13] Yang Y., Chang W. (2024). Analysis of Spatial and Temporal Distribution and Changes in Extreme Climate Events in Northwest China from 1960 to 2021: A Case Study of Xinjiang. Sustainability.

[14] Liu F., You Z., Yang X., Wang H. (2018). Macro-micro degradation process of fly ash concrete under alternation of freeze-thaw cycles subjected to sulfate and carbonation. Constr. Build. Mater..

[15] Zhao R., Yuan Y., Cheng Z., Wen T., Li J., Li F., Ma Z.J. (2019). Freeze-thaw resistance of Class F fly ash-based geopolymer concrete. Constr. Build. Mater..

[16] Ma Z., Zhu F., Ba G. (2019). Effects of freeze-thaw damage on the bond behavior of concrete and enhancing measures. Constr. Build. Mater..

[17] Zhang P., Cong Y., Vogel M., Liu Z., Müller H.S., Zhu Y., Zhao T. (2017). Steel reinforcement corrosion in concrete under combined actions: The role of freeze-thaw cycles, chloride ingress, and surface impregnation. Constr. Build. Mater..

[18] Zhang S., Chen B., Tian B., Lu X., Xiong B. (2022). Effect of Fly Ash Content on the Microstructure and Strength of Concrete under Freeze–Thaw Condition. Buildings.

[19] Zhang S., Tian B., Chen B., Lu X., Xiong B., Shuang N. (2022). The Influence of Freeze–Thaw Cycles and Corrosion on Reinforced Concrete and the Relationship between the Evolutions of the Microstructure and Mechanical Properties. Materials.

[20] Papadakis V.G. (2000). Effect of supplementary cementing materials on concrete resistance against carbonation and chloride ingress. Cem. Concr. Res..

[21] Heidari M., Salaudeen S., Arku P., Acharya B., Tasnim S., Dutta A. (2021). Development of a mathematical model for hydrothermal carbonization of biomass: Comparison of experimental measurements with model predictions. Energy.

[22] Van den Heede P., De Belie N. (2015). Durability based life cycle assessment of concrete with supplementary cementitious materials exposed to carbonation. Int. Conf. Sustain. Struct. Concr. Proc..

[23] Heede P.V.D., De Belie N. (2014). A service life based global warming potential for high-volume fly ash concrete exposed to carbonation. Constr. Build. Mater..

[24] Li S., Chen Y., Tang C., Wang J., Liu R., Wang H. (2022). Experimental and Theoretical Study on Carbonization Coefficient Model of NS/SAP Concrete. Buildings.

[25] Kruse A., Grandl R. (2015). Hydrothermal: 3. Kinetic model, Chemie-Ingenieur-Technik. Chem. Ing. Tech..

[26] Tan Y., Ma C., Zhao B., Xiong W., Chen X., Yu J. (2023). Study and Microanalysis on the Effect of the Addition of Polypropylene Fibres on the Bending Strength and Carbonization Resistance of Manufactured Sand Concrete. Polymers.

[27] Ou G., Xin Z., Chengkai Z. (2015). Prediction models of the carbonization depth of recycled concrete. Zhongguo Kuangye Daxue Xuebao/J. China Univ. Min. Technol..

[28] Zhang J.-S., Cheng M., Zhu J.-H. (2020). Carbonation Depth Model and Prediction of Hybrid Fiber Fly Ash Concrete. Adv. Civ. Eng..

[29] Chen Z., Hu Y.C., Zhao Y.F., Yu B. (2019). Multi—Factor Computation Model of Concrete Carbonation Depth Based on Material Parameters in Standard Carbonization Environment. Bull. Chin. Ceram. Soc..

[30] Li G.F., Shen X.D. (2019). A Study of the deterioration law and mechanism of aeolian-sand powder concrete in the coupling environments of freeze-thaw and carbonization. J. Ceram. Soc. Jpn..

[31] Yang L., Zhu Z., Sun H., Huo W., Zhang J., Wan Y., Zhang C. (2023). Durability of waste concrete powder-based geopolymer reclaimed concrete under carbonization and freeze–thaw cycles. Constr. Build. Mater..

[32] Niu J.G., Yan L., Zhai H.T. (2013). Study on the influence of freeze-thaw on the carbonation property of fly ash concrete. Appl. Mech. Mater..

[33] Li Y., Wang R., Zhao Y. (2017). Effect of coupled deterioration by freeze-thaw cycle and carbonation on concrete produced with coarse recycled concrete aggregates. J. Ceram. Soc. Jpn..

[34] Zhang R., Du L., Li Z., Lin P., Cao F. (2023). Design Method For Cemented Sand and Gravel Mix Proportion Using Jaw-Crushed Material. J. Eng. Sci. Technol. Rev..

[35] (2009). Standard for Test Methods of Long-Term Performance and Durability of Ordinary Concrete.

[36] Zhang D., Wang Y., Ma M., Guo X., Zhao S., Zhang S., Yang Q. (2022). Effect of Equal Volume Replacement of Fine Aggregate with Fly Ash on Carbonation Resistance of Concrete. Materials.

[37] Li B., Tian Y., Zhang G., Liu Y., Feng H., Jin N., Jin X., Wu H., Shao Y., Yan D. (2024). Author Correction: Comparison of detection methods for carbonation depth of concrete. Sci. Rep..

[38] Niu D., Xiao Q., Zhu W. (2012). Concrete damage and neutralization under coupling effect of carbonation and freeze-thaw cycles. J. Wuhan Univ. Technol. Mater. Sci. Ed..

[39] Kuosa H., Ferreira R., Holt E., Leivo M., Vesikari E. (2014). Effect of coupled deterioration by freeze–thaw, carbonation and chlorides on concrete service life. Cem. Concr. Compos..

[40] Li Y., Wang R., Li S., Zhao Y., Qin Y. (2018). Resistance of recycled aggregate concrete containing low- and high-volume fly ash against the combined action of freeze–thaw cycles and sulfate attack. Constr. Build. Mater..

[41] Rao M.J., Dong Y., Yang H.Q., Li M.X., Yu Z. (2016). Influence of carbonation and freeze-thaw on macro-properties of concrete. J. Phys. Conf. Ser..

[42] Zhang K., Zhou J., Yin Z. (2021). Experimental study on mechanical properties and pore structure deterioration of concrete under freeze–thaw cycles. Materials.

[43] Chen B., Guan B., Lu X., Tian B., Li Y. (2022). Thermal conductivity evolution of early-age concrete under variable curing temperature: Effect mechanism and prediction model. Constr. Build. Mater..

[44] Zhou H., Mi T., Zhao C., Liang Z., Fang T., Wang F., Zhou Y. (2024). Identification Model of Fault-Influencing Factors for Dam Concrete Production System Based on Grey Correlation Analysis. Appl. Sci..

[45] Zhou M., Dong W. (2023). Grey relativity correlations between the pore structures and compressive strength of aeolian sand concrete undergoing carbonation and freeze-thaw cycles. J. Build. Eng..

[46] Tian L., Chen J., Zhao T. (2012). Durability of lining concrete of subsea tunnel under combined action of freeze-thaw cycle and carbonation. J. Wuhan Univ. Technol. Mater. Sci. Ed..

